# Malaria genomics: tracking a diverse and evolving parasite population

**DOI:** 10.1093/inthealth/ihv007

**Published:** 2015-02-26

**Authors:** Dominic Kwiatkowski

**Affiliations:** Wellcome Trust Sanger Institute, Hinxton, Cambridge, CB10 1SA and Oxford University, Henry Wellcome Building for Molecular Physiology, Old Road Campus, Headington, Oxford, OX3 7BN, UK

**Keywords:** Artemisinin resistance, Genomic epidemiology, Malaria, *Plasmodium falciparum*

## Abstract

Malaria parasites are continually evolving to evade the immune system and human attempts to control the disease. To eliminate malaria from regions where it is deeply entrenched we need ways of monitoring what is going on in the parasite population, detecting problematic changes as soon as they arise, and executing a prompt and effective response based on a deep understanding of this natural evolutionary process. Powerful new tools to address this problem are emerging from the fast-growing field of genomic epidemiology, driven by new sequencing technologies and computational methods that allow parasite genome variation to be studied in much greater detail and in many more samples than was previously considered possible. These new tools will provide a deep understanding of what is going on in the parasite population, generating actionable knowledge for strategic planning of control interventions, for monitoring their effects and steering them for greatest impact, and for raising the alert if things start to go wrong.

Over a century ago, it was discovered that human malaria is caused by several distinct species of *Plasmodium* that cause different patterns of clinical illness. The discovery was made by microscopic examination of blood films, and this method of species identification has remained the mainstay of malaria epidemiology to the present day. However, if we examine parasite diversity at a deeper level, beyond the level of resolution achievable by microscopy, it turns out that each *Plasmodium* species is made up of a genetically heterogeneous population of parasites that is continually changing.^1^ These changes partly reflect population flux (i.e., the ebb and flow of different genetic types due to the movements of humans and mosquitoes) and other demographic factors. Other changes are due to evolutionary adaptation, e.g., parasite genes involved in erythrocyte invasion and endothelial cytoadherence maintain extraordinarily high levels of diversity to evade host defence mechanisms, an evolutionary arms race that has ancient origins and is still ongoing.^2,3^

Malaria control interventions cause the parasite population to change but not always in the way that was intended. The use of antimalarial drugs over the past 60 years has saved millions of lives but can also be viewed as an evolutionary experiment with a disastrous result as *P. falciparum* is now resistant to chloroquine and pyrimethamine almost everywhere, and resistance to artemisinin, the current frontline drug, is spreading through Southeast Asia.^4–6^ Evidently, malaria parasites have massive capacity for evolutionary adaptation, and any control strategy that involves scaling-up the use of antimalarial drugs or introducing a vaccine must prepare to encounter new forms of resistance. To eliminate malaria from regions where it is deeply entrenched, we need ways of monitoring what is going on in the parasite population, detecting problematic changes as soon as they arise, and executing a prompt and effective response based on a deep understanding of this natural evolutionary process.

Powerful new tools to address this problem are emerging from the fast-growing field of genomic epidemiology, driven by new sequencing technologies and computational methods that allow parasite genome variation to be studied in much greater detail (and in many more samples) than was previously considered possible. This revolution in parasite genetics began with construction of a reference genome sequence for *P. falciparum*, which revealed all of the genes that encode the protein building blocks of this parasite, numbering more than 5000, and enabled the first systematic efforts to characterise common patterns of variation throughout the genome.^7^ Another crucial advance was the advent of next-generation sequencing technologies and the development of practical methods to sequence the parasite genome directly from clinical blood samples.^1^ Large collaborative initiatives, such as the MalariaGEN *Plasmodium falciparum* Community Project and the Pf3k Consortium have been instrumental in providing researchers in malaria-endemic countries with access to these new technologies and in developing data-sharing policies that encourage researchers to share genome variation data at an early stage.^8,9^ As a result, a huge amount of data on *P. falciparum* genome variation is now openly available through easily accessible web applications, including sequencing data on thousands of samples from around the world and allele frequency data on almost a million single nucleotide polymorphisms (SNPs).

We still have only a partial view of the landscape of *P. falciparum* genome variation, based primarily on SNPs, but omitting regions of the genome that are difficult to analyse with current technologies, and also omitting other classes of polymorphism such as small insertions and deletions (indels), copy number variants, large structural variants and the hypervariable *var*, *rifin* and *stevor* gene families. The goal of the Pf3k Consortium is to lay out the whole landscape of *P. falciparum* genome variation by progressive technological and analytical improvements that will ultimately allow detailed characterisation of all classes of polymorphism in large epidemiological surveys.^8^ However, much useful information can be gleaned from the available data, e.g., about the distribution and flow of drug resistance, candidate vaccine polymorphisms, genes that have undergone recent evolutionary selection and genetic differences between parasite populations in different locations.^1,2,10–14^ Simple tools are emerging from these complex analyses, such as genetic assays that define the likely geographical origin of a malaria infection.^15^ The power of these tools will increase as the amount of genome variation data grows in scale and resolution.

The spread of artemisinin resistant *P. falciparum* across Southeast Asia provides a good illustration of the epidemiological insights that can come from genome variation data and their practical relevance for malaria control. Important clues about the genetic basis of artemisinin resistance came from genome sequencing of a laboratory strain of *P. falciparum* with experimentally-induced resistance,^16^ and also from genome-wide analyses of evolutionary selection, phenotypic association and population genetics in clinical samples.^10,16–18^ These genome-wide analyses pointed to a specific molecular marker of artemisinin resistance, the propeller domain of *kelch13*, a gene that had previously gone unnoticed and whose precise biological function remains unknown.^16^ Further epidemiological and experimental studies have provided compelling evidence that artemisinin resistance is caused by any one of multiple independent mutations at this specific locus in the *P. falciparum* genome.^6,14,19–21^ This remarkable discovery provides a scientific foundation for investigating the mechanism of resistance as well as some surprising new insights into the way that artemisinin resistance is spreading.

A critical finding is that the geographical diffusion of artemisinin resistance is driven largely by the emergence of new *kelch13* mutations, which have occurred independently in multiple locations, rather than by the spread of a single mutation. Over 20 independent *kelch13* propeller mutations have now been associated with artemisinin resistance and the most common of these, C580Y, has multiple origins.^6,14,19^ Strong founder effects can be observed as new mutations spread through the parasite population, analogous to the clonal expansion of cancer cells that acquire driver mutations.^10,19^ Why have so many resistance-causing mutations emerged independently in this particular geographical region of Southeast Asia? A recent multi-centre clinical study, in which *P. falciparum* genome sequencing was performed on more than 1000 cases, has identified genetic markers of predisposition to artemisinin resistance, i.e., markers of the risk that an artemisinin-sensitive parasite will acquire a resistance-causing *kelch13* mutation.^19^ These new markers essentially define the geographical boundaries of artemisinin resistance in Southeast Asia and raise fundamental questions about how resistance emerges and spreads, e.g., whether *kelch13* mutations need to occur on a particular genetic background in order to cause resistance or to ensure their biological fitness.

An important practical question is whether *kelch13* mutations are a reliable indicator of artemisinin resistance outside Southeast Asia. There are growing reports of *kelch13* propeller mutations in Africa whose clinical significance remains to be defined, but the available evidence suggests that they are less likely to be associated with artemisinin resistance. This highlights the importance of understanding how *kelch13* mutations interact with their genetic background.^6,22,23^ Efforts to develop reliable markers of resistance need to take account of the evolving nature of the artemisinin resistance phenotype in Southeast Asia: it began as delayed parasite clearance, but there are increasing reports of complete treatment failure, which may affect the likelihood of spread to Africa. Also, there is growing resistance to partner drugs used in artemisinin combination therapy such as piperaquine. Moving forward, there is a strong case for collecting clinical data on resistance together with parasite sequencing data whenever this is practically feasible, both in Southeast Asia and in other parts of the world, and continually updating genetic analyses to keep track of an epidemiological phenomenon that remains poorly understood and is rapidly evolving.

Over the past decade, *Plasmodium* genome sequencing has been transformed from an activity restricted to world-leading genome centres into a relatively well-defined set of procedures and pipelines that are potentially accessible to all malaria researchers. Assembly of the full genome sequence of a parasite remains challenging and expensive with current technologies, but reliable data on most genes can now be feasibly generated on tens of thousands of samples with continually falling unit costs. The main challenge is not so much in generating data but in analysing and making sense of it; and the useful information that can be obtained from an individual sample is greatly increased if it is analysed in the context of thousands of other samples. As with any other type of epidemiological data, analyses become with larger sample sizes and higher quality of metadata, such as reliable spatiotemporal information and clinical phenotypes. Information systems are needed for integrating and analysing data on a global scale, with simple mechanisms to allow clinicians, researchers and malaria control teams to submit samples or data and to get back useful information in a timely and comprehensible manner. The intention is not to replace blood film microscopy or rapid diagnostic tests, which are clearly more appropriate for decision making at the point of care. The need is to gain actionable knowledge for strategic planning of control interventions based on a deep understanding of what is going on in the parasite population, for monitoring the effects of interventions and steering them for greatest impact, and for raising the alert if things start to go wrong.

## References

[1] Manske M, Miotto O, Campino S (2012). Analysis of *Plasmodium falciparum* diversity in natural infections by deep sequencing. Nature.

[2] Amambua-Ngwa A, Tetteh KK (2012). Manske M et al. Population genomic scan for candidate signatures of balancing selection to guide antigen characterization in malaria parasites. PLoS Genet.

[3] Claessens A, Hamilton WL, Kekre M (2014). Generation of antigenic diversity in *Plasmodium falciparum* by structured rearrangement of Var genes during mitosis. PLoS Genet.

[4] Naidoo I, Roper C (2013). Mapping ‘partially resistant’, ‘fully resistant’, and ‘super resistant’ malaria. Trends Parasitol.

[5] Sá JM, Chong JL, Wellems TE (2011). Malaria drug resistance: new observations and developments. Essays Biochem.

[6] Ashley EA, Dhorda M, Fairhurst RM (2014). Spread of artemisinin resistance in *Plasmodium falciparum* malaria. N Engl J Med.

[7] Gardner MJ, Hall N, Fung E (2002). Genome sequence of the human malaria parasite *Plasmodium falciparum*. Nature.

[8] Pf3k Consortium www.malariagen.net/apps/pf3k/.

[9] MalariaGEN Plasmodium falciparum Community Project www.malariagen.net/apps/pf/.

[10] Miotto O, Almagro-Garcia J, Manske M (2013). Multiple populations of artemisinin-resistant *Plasmodium falciparum* in Cambodia. Nat Genet.

[11] Ocholla H, Preston MD, Mipando M (2014). Whole-genome scans provide evidence of adaptive evolution in Malawian *Plasmodium falciparum* isolates. J Infect Dis.

[12] Mobegi VA, Duffy CW, Amambua-Ngwa A (2014). Genome-wide analysis of selection on the malaria parasite *Plasmodium falciparum* in West African populations of differing infection endemicity. Mol Biol Evol.

[13] Nwakanma DC, Duffy CW, Amambua-Ngwa A (2014). Changes in malaria parasite drug resistance in an endemic population over a 25-year period with resulting genomic evidence of selection. J Infect Dis.

[14] Takala-Harrison S, Jacob CG, Arze C (2015). Independent emergence of artemisinin resistance mutations among *Plasmodium falciparum* in Southeast Asia. J Infect Dis.

[15] Preston MD, Campino S, Assefa SA (2014). A barcode of organellar genome polymorphisms identifies the geographic origin of *Plasmodium falciparum* strains. Nat Commun.

[16] Ariey F, Witkowski B, Amaratunga C (2014). A molecular marker of artemisinin-resistant *Plasmodium falciparum* malaria. Nature.

[17] Takala-Harrison S, Clark TG, Jacob CG (2013). Genetic loci associated with delayed clearance of *Plasmodium falciparum* following artemisinin treatment in Southeast Asia. Proc Natl Acad Sci USA.

[18] Cheeseman IH, Miller BA, Nair S (2012). A major genome region underlying artemisinin resistance in malaria. Science.

[19] Miotto O, Amato R, Ashley EA (2015). Genetic architecture of artemisinin-resistant *Plasmodium falciparum*. Nat Genet.

[20] Ghorbal M, Gorman M, Macpherson CR (2014). Genome editing in the human malaria parasite *Plasmodium falciparum* using the CRISPR-Cas9 system. Nat Biotechnol.

[21] Straimer J, Gnadig NF, Witkowski B (2015). K13-propeller mutations confer artemisinin resistance in *Plasmodium falciparum* clinical isolates. Science.

[22] Kamau E, Campino S, Amenga-Etego L (2014). K13-propeller polymorphisms in *Plasmodium falciparum* parasites from sub-Saharan Africa. J Infect Dis.

[23] Taylor SM, Parobek CM, DeConti DK (2015). Absence of putative artemisinin resistance mutations among *Plasmodium falciparum* in Sub-Saharan Africa: A molecular epidemiologic study. J Infect Dis.

